# FIGO 2023 endometrial staging: a leap of faith into the new “prognostic based’ rather than “anatomical based” staging—too fast too furious??

**DOI:** 10.1007/s00432-024-05739-w

**Published:** 2024-05-11

**Authors:** Karthik Chandra Bassetty, Dimpy Begum, Debabrata Barmon, Upasana Baruah, Sakshi Gupta, Mahendra Kumar, Jyotiman Nath, Duncan Khanikar, Mouchumee Bhattacharyya, P. S. Roy

**Affiliations:** https://ror.org/018dzn802 grid.428381.4 0000 0004 1805 0364 Dr. Bhubaneswar Borooah Cancer Institute, Guwahati, Assam India

**Keywords:** Endometrial cancer, FIGO 2023, Stage shift, Prognosis, LMIC

## Abstract

**Background:**

In 2023 FIGO revised the endometrial cancer staging system after 13 years. There is a lacuna of data regarding the performance and practicality of the revised 2023 FIGO staging schema for endometrial cancer from Low Middle-Income Countries (LMIC).

**Objective:**

To estimate the shift of stage and adjuvant management of endometrial cancer based on the FIGO 2023 system compared to the FIGO 2009 system and assess the predictive potential of the FIGO 2023 system.

**Material and methods:**

A retrospective study was conducted from 1st January 2017 to 31st December 2022. All patients with endometrial cancer were staged according to the FIGO 2023 and FIGO 2009 staging system. Follow-up of patients was done to determine recurrence.

**Results:**

A total of 152 patients were included. Aggressive histology was seen in 66 (45%) patients. Eighteen (11%) had subserosal involvement. Substantial LVSI was noted in 23 (15%) of patients. Twenty-four (47%) patients of FIGO 2009 Stage IA and 26 patients (63%) of FIGO 2009 Stage IB were upstaged. Eleven (50%) patients of FIGO 2009 Stage IIIA were down staged to IA3. Overall 23 patients (15%) had a shift of stage. Fifteen out of 152 patients (15%) would have had a possible risk stratification change which would imply 23 patients (15%) would have needed a more radical treatment. Molecular classification was done in 32 patients; however, only 2 patients could afford POLE testing. Kaplan–Meier curves showed significant PFS differences in FIGO 2009 Stage IB and Stage IIIA when restaged according to the FIGO 2023 system.

**Conclusion:**

The FIGO 2023 endometrial staging is a more robust prognosticator; however, the practicality of molecular classification in LMICs is still a distant dream.

## Introduction

Staging systems are the backbone of any cancer treatment. They are meant to be valid, reliable and practical (Odicino et al. 2008). Stage is a strong predictor of patient prognosis. The main bodies for cancer staging include the American Joint Committee on Cancer (AJCC) and the Union for International Cancer Control (UICC) (Brierley. 2006). Concerning gynaecological cancer, The International Federation of Gynaecology and Obstetrics (FIGO) takes centre stage and a collaborative arrangement between the three staging systems ensures synchrony with changes in FIGO staging manifesting in the AJCC and UICC versions (Greene and Sobin 2009).

The earlier anatomy-based staging was the norm earlier, however, over the years staging has included histological prognostic factors, biomarkers and molecular data as evident in breast, head-neck and prostate cancers (Odicino et al. 2008; Amin et al. 2017). Keeping with this trend a radical shift in endometrial cancer (EC) staging was formulated by FIGO in June 2023 (Berek 2023). The changes in the FIGO 2023 endometrial staging system compared to the FIGO 2009 endometrial staging system are described in Table 1. It has created quite a stir in the oncology fraternity on its inception. Keeping this background in mind, we devised a retrospective study to study the impact of the FIGO 2023 endometrial staging system compared to its predecessor FIGO 2009 endometrial staging system.

**Table 1:** Differences between FIGO 2009 and FIGO 2023 endometrial staging systems

FIGO 2009		FIGO 2023	
IA	Tumour confined to endometrium or < 50% MI	IA1	NAH confined to polyp or endometrium
IB	Tumour ≥ 50% MI confined to the uterus	IA2	NAH with MI < 50% + LVSI absent
		IA3	NAH involvement of uterus and U/L ovary*
		IB	NAH MI ≥ 50% + LVSI absent
		IC	AH -polyp or endometrium
II	Cervical stromal invasion(CSI)	IIA	NAH + CSI
		IIB	NAH + LVSI
		IIC	AH + MI
IIIA	Serosa /adnexa involvement	IIIA1	Ovarian and fallopian tube involvement and not meeting IA3 criteria
		IIIA2	Sub serosa or serosa involvement
IIIB	Vagina/parametrium involvement	IIIB1	Vagina/parametrium involvement
		IIIB2	Pelvic peritoneum involvement
IIIC1	Pelvic LN metastasis	IIIC1i	Pelvic nodes micrometastasis#
		IIIC1ii	Pelvic node macrometastasis
IIIC2	Para-aortic LN metastasis	IIIC2i	Para-aortic nodes micrometastasis
		IIIC2ii	Para-aortic nodes macrometastasis
IVA	Bladder /bowel mucosa involvement	IVA	Bladder/bowel mucosa involvement
IVB	Distant metastasis	IVB	Abdominal metastasis beyond the pelvis
		IVC	Distant metastasis

*MI* myometrial invasion, *NAH* non-aggressive histology, i.e. Grades I and II endometrioid histology, *LVSI* lymphovascular space invasion, *AH* aggressive histology, i.e. Grade III endometrioid, serous, clear cell, mesonephric type and other histology

*Criteria for Stage IA3—cases of low-grade endometrioid endometrial cancer with: (1) no more than superficial myometrial invasion is present (< 50%); (2) the absence of substantial LVSI; (3) the absence of additional metastases; and (4) the ovarian tumour is unilateral, limited to the ovary, without capsule invasion/breach (equivalent to pT1a)

^#^Micrometastasis > 0.2–2 mm in size and macrometastasis > 2 mm in size

## Objective

### Primary objective

To determine the percentage change in stage and risk stratification of patients with endometrial cancer from the FIGO 2009 endometrial staging system to the FIGO 2023 endometrial staging system.

### Secondary objective

To determine the possible impact of the stage shift on the management of patients and assess the prognostic impact of the FIGO 2023 endometrial staging system.

## Materials and methods

### Study design

All patients who were diagnosed with EC and initially underwent surgery at the Dr. Bhubaneswar Borooah Cancer Institute (BBCI), Guwahati, Assam, India between 1st January 2017 and 31st December 2022 were included in the study. All patients underwent surgical staging which included a total hysterectomy and bilateral salpingo-oophorectomy. Lymph node dissection was conducted in all patients except in patients with Stage IA—Grades I and II endometrioid endometrial cancer with a tumor size of less than 2 cm. Patients who received neoadjuvant chemotherapy, had recurrent endometrial cancer, operated outside BBCI, had multiple cancers, had no available formalin fixed paraffin embedded samples and whose tissue samples were inadequate were excluded. We had incorporated immunohistochemistry testing for MMR proteins (mismatch repair proteins MLH 1, MSH 2, MSH 6 and PMS 2) and p53 from 1st January 2023. POLE testing is not done in our institute due to the cost factor and depending on the affordability of the patient it was outsourced. The patients were divided into four groups—POLEmut, MMR-D, p53abn and NSMP. In the event of a patient testing positive for multiple IHCs the algorithm of ProMisE study was followed (Kommoss et al. 2018). Patients who were treated before molecular testing were classified without the molecular testing. All the histopathology reports were reviewed by two expert oncopathologists and were reported as per the 2020 World Health Organization tumor classification criteria which included the histopathological type, extent of LVSI and size of lymph node metastasis. The histopathological slides were reviewed where a discrepancy or doubt occurred regarding the findings. Demographic and clinical data were obtained retrospectively from the electronic medical registry (EMR). In this study the patients who were previously stratified based on FIGO 2009 endometrial staging system were restratified based on FIGO 2023 endometrial staging system. Upstaging was described as the reclassification to a higher group and downstaging was the reclassification to the lower group in the FIGO 2023 staging system compared to the FIGO 2009 staging system.

## Operational definitions as per the FIGO 2023 endometrial staging system (Berek 2023)

1. Histology:
  Non-aggressive histological types are composed of low-grade (Grades 1 and 2) endometrioid cancers, while aggressive histological types are composed of high-grade endometrioid cancer (Grade 3), serous carcinoma, clear cell carcinoma, mixed carcinoma, undifferentiated variant, carcinosarcoma and mesonephric-like and gastrointestinal type mucinous carcinomas.
2. Lymphovascular space invasion (LVSI):
  Involvement of ≥ 5 vessels is considered as substantial LVSI and less than 5 vessels is considered as focal LVSI.
3. Involvement of sub-serosa:
  As per the ISGYP recommendations (Singh 2019) uterine serosa involvement is defined as a tumour reaching submesothelial fibro connective tissue or the mesothelial layer, regardless of whether tumour cells may or may not be present on the serosal surface of the uterus.
4. Adnexal involvement:
  The 2023 FIGO staging for endometrial carcinoma assigns the category of Stage IA3 when the following criteria are met in a low-grade endometrioid endometrial cancer:

1. No more than superficial myometrium invasion is present (< 50%).
2. The absence of substantial LVSI.
3. The absence of additional metastases.
4. The ovarian tumour is unilateral, limited to the ovary, without capsule invasion/breach (equivalent to pT1a).

The cases not fulfilling these criteria were interpreted as extensive spread of the endometrial carcinoma to the ovary (Stage IIIA1).

All patients underwent risk stratification as per standard guidelines existing (Concin et al. 2021; Colombo et al. 2016; Endometrical Cancer 2024) and received appropriate adjuvant radiotherapy and chemotherapy. We also studied the possible change in risk stratification when patients were stratified as per the ESMO 2022 risk stratification system (Endometrical Cancer 2024) using the FIGO 2023 staging system.

### Statistical analysis

Demographic and clinical data were expressed as the number of patients and percentage (%). Progression-free survival (PFS) was calculated from the date of completion of treatment to the date of recurrence or last contact. The Kaplan–Meier method was used to analyze the PFS and the log-rank test was used to evaluate the between-group differences in PFS. The cutoff p value for statistical significance was < 0.05. SPSS version 29.0 was used for statistical analysis.

## Results

**Fig. 1:**
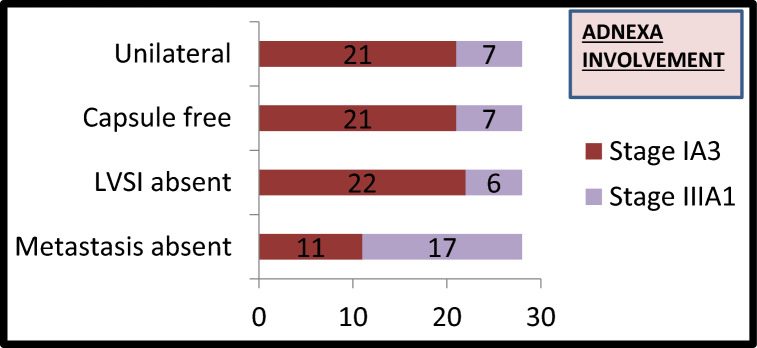
Bar diagram depicting the 28 women with adnexal involvement with characteristics about unilaterality, capsule involvement, LVSI and presence of metastasis

A total of 152 patients were included in the study. The median age of the patients was 52 years (range 23–84 years). The majority of patients (144, 95%) underwent open surgery whereas eight patients (5%) underwent minimally invasive surgery. Table 2 shows the histo-pathological data of all 152 patients. The majority (57%) had non-aggressive histology. Substantial LVSI was noted in 15% of cases. Out of 28 women who had adnexal involvement with low-grade endometrioid carcinoma only eleven patients fulfilled the criteria to be reclassified as Stage IA3 as shown in Fig. 1. Metastasis to the pelvic nodes was seen in 13% of patients whereas para-aortic nodal metastasis was documented in 3% of cases.

**Table 2:** Characteristics of 152 patients with endometrial cancer

Category	*N* = 152	%
*Histology*:		
A. *Non-aggressive histology:*		
i. Endometriod Grade I	61	41%
ii. Endometriod Grade II	25	16%
B. *Aggressive histology*:		
i. Endometrioid Grade III	36	23%
ii. Serous	22	14%
iii. Clear cell	6	4%
iv. Carcinosarcoma	2	2%
*Myometrium invasion*		
a. Limited to endometrium	12	8%
b. Invasion < 50%	62	40%
c. Invasion ≥ 50%	78	52%
*Cervical stromal involvement*		
No invasion	141	93%
Cervical stromal	11	7%
*Serosa involvement*		
a. No invasion	124	81%
b. Sub serosa	18	12%
c. Serosa	10	7%
*Lymphovascular space invasion* (*LVSI*)		
a. Absent	127	84%
b. Focal	2	1%
c. Substantial	23	15%
*Peritoneal metastasis*		
a. POD deposits	8	5%
b. Extra pelvic peritoneum involvement	1	0.6%
c. Omentum	4	3%
*Nodal involvement*		
*Pelvic lymph involvement*		
Not done	7	5%
Positive	20	13%
Negative	125	82%
*Para-aortic involvement*		
Not done	58	38%
Positive	5	3%
Negative	89	59%

**Fig. 2:**
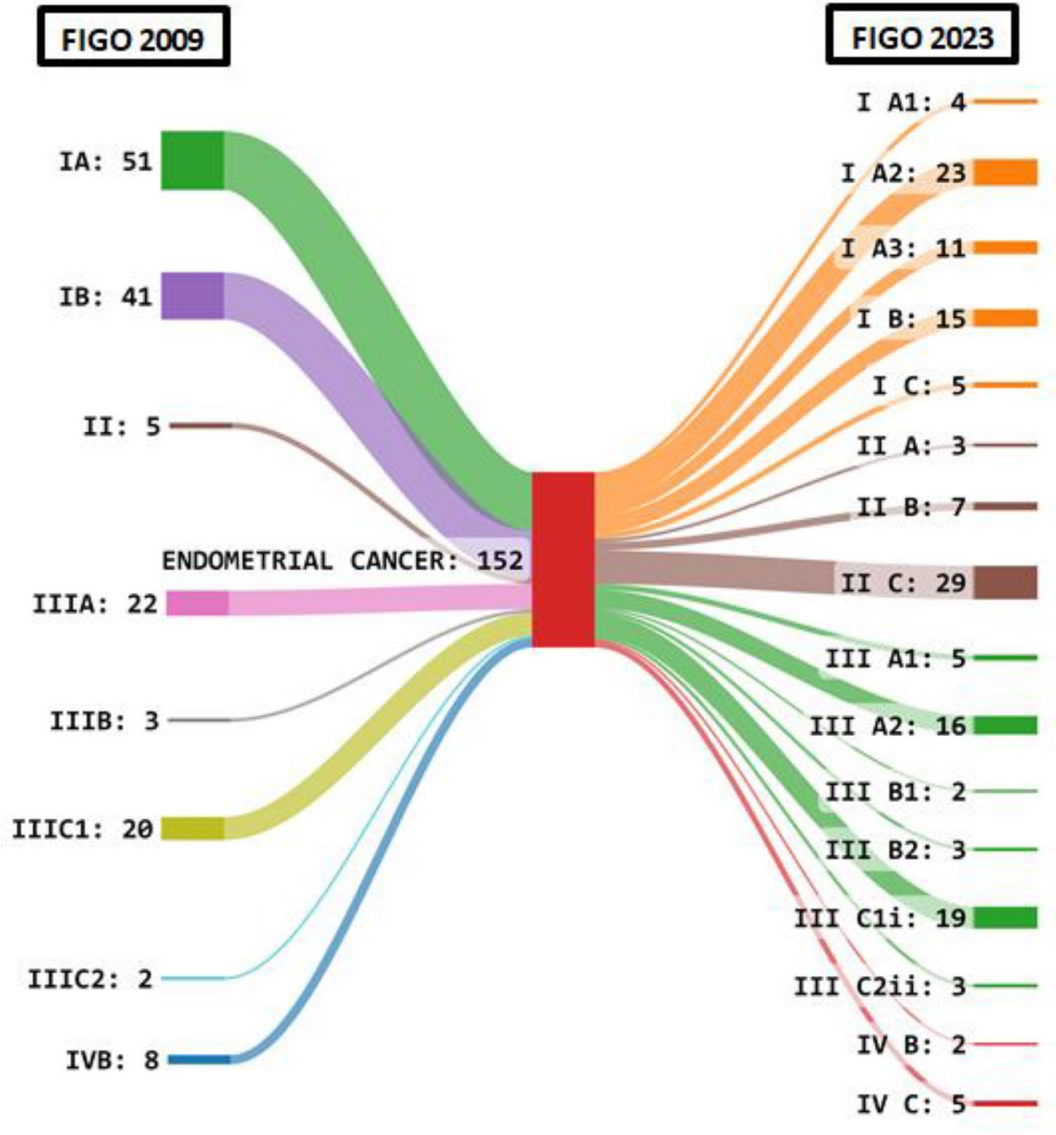
Sankey diagram showing the difference in staging of patients as per the FIGO 2009 and FIGO 2023 endometrial staging systems

Figure 2 shows the Sankey diagram depicting the change in staging of patients as per the FIGO 2009 and FIGO 2023 endometrial staging systems. Due to reclassification number of patients in Stage I decreased from 92 to 38. There was a sharp increase in Stage II patients (39) in the 2023 system and a decline in Stage III with a dramatic decrease in number of patients with Stage IIIA from 22 to 5 patients. Overall 35 out of 152 (23%) patients had a change in the stage.

Table 3 shows the stage shift concerning the individual sub-stages of FIGO 2009 and FIGO 2023 endometrial staging systems. It was seen that 24 out of 51 patients (47%) were staged in Stage IA whereas 26 out of 41 patients (67%) in Stage IB were upstaged. In Stage IIIA 11 out of 22 patients (50%) were down staged to IA3 whereas in Stage IVB 5 out of 8 patients (62.5%) were upstaged.

**Table 3:** Stage shifts in the study cohort of 152 patients according to FIGO 2009 and FIGO 2023 staging systems

Stage FIGO 2009	Same stage FIGO 2023	Upstaged FIGO 2023	Downstaged FIGO 2023
IA (51)	IA1 (4)	IC (6)	
	IA2 (23)	IIC (16)	
		IIIA2 (2)	
IB (41)	IB (15)	IIB (6)	
		IIC (11)	
		IIIA2 (9)	
II (5)	IIA (2)		
	IIB (1)		
	IIC (2)		
IIIA (22)	IIIA1 (5)	IIIB2 (1)	IA3 (11)
	IIIA2 (5)		
IIIB (3)	IIIB1(2)	IVB (1)	
IIIC1 (20)	IIIC1ii (19)		IIIBS (1)
IIIC2 (2)	IIIC2ii (2)		
IVB (8)	IVB (1)	IVC (5)	IIIB2 (1)
			IIIC2ii (1)

**Fig. 3:**
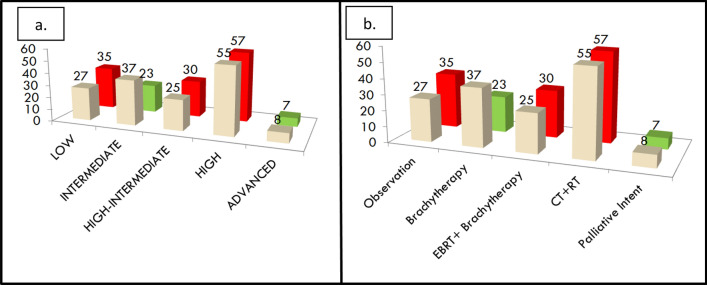
**a** The possible change in risk stratification between FIGO 2009 and FIGO 2023 staging systems. **b** The possible change in adjuvant treatment that would have been needed by patients when restaged as per FIGO 2023 staging system. The cream shade refers to the FIGO 2009 system and the green and red refer to the FIGO 2023 system wherein green depicts a decrease in numbers and red depicts the increase in numbers

Figure 3a shows the possible shift in the risk stratification category and Fig. 3b shows the possible change in adjuvant treatment that would have resulted by the application of the ESMO 2022 risk scoring and FIGO 2023 staging system. It was observed that 15 out of 152 patients (10%) would have had a change in the risk stratification score as shown in Fig. 3a. In Fig. 3b as shown 7 out of 152 patients (5%) would have been undertreated whereas 23 out of 152 patients (15%) would have been overtreated.

Table 4 shows the risk stratification shift between FIGO 2009 and FIGO 2023 staging systems using the ESMO 2022 stratification system. It is observed that 16 out of 37 patients (43%) had increased risk stratification in the FIGO 2009 intermediate risk strata group whereas 10 out of 55 patients (18%) had decreased risk stratification in FIGO 2009 high-risk strata group.

**Table 4:** Risk stratification shift in the study cohort of 152 patients according to the FIGO 2009 and FIGO 2023 staging system using the ESMO 2022 risk stratification system

Risk strata as per FIGO 2009	“Same” risk strata as per FIGO 2023	“Upstaged” risk strata as per FIGO 2023	“Down staged” risk strata as per FIGO 2023
Low (27)	Low (27)		
Intermediate (37)	Intermediate (21)	High Intermediate (9) High (7)	
High Intermediate (25)	High intermediate (21)	High (4)	
High (55)	High (45)		Low (8) Intermediate (2)
Advanced (8)	Advanced (7)		High (1)

**Fig. 4:**
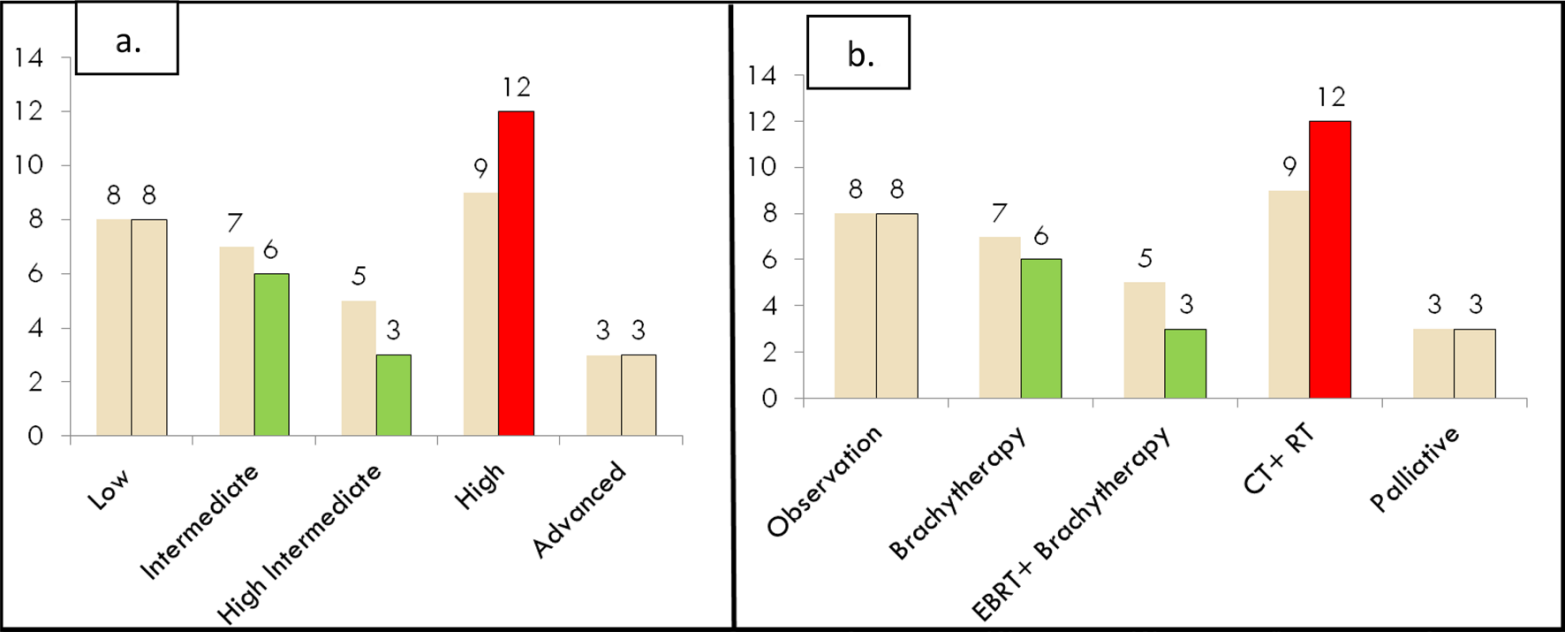
**a** The impact of molecular profiling on risk stratification. **b** The change in adjuvant treatment based on the risk strata

When we analyzed the molecular profiling of 32 patients in the 2023 as shown in Fig. 4a and b it was observed only two patients could afford POLEmut testing and both of the reports were negative for POLE mutation. It was observed that 3 out of 32 patients (9%) would have had their risk stratification strata changed as seen with the high-risk strata group increasing from 9 to 12 cases. We treated those patients with sequential chemotherapy and radiation therapy as dictated by the high-risk group management guidelines.

**Fig. 5:**
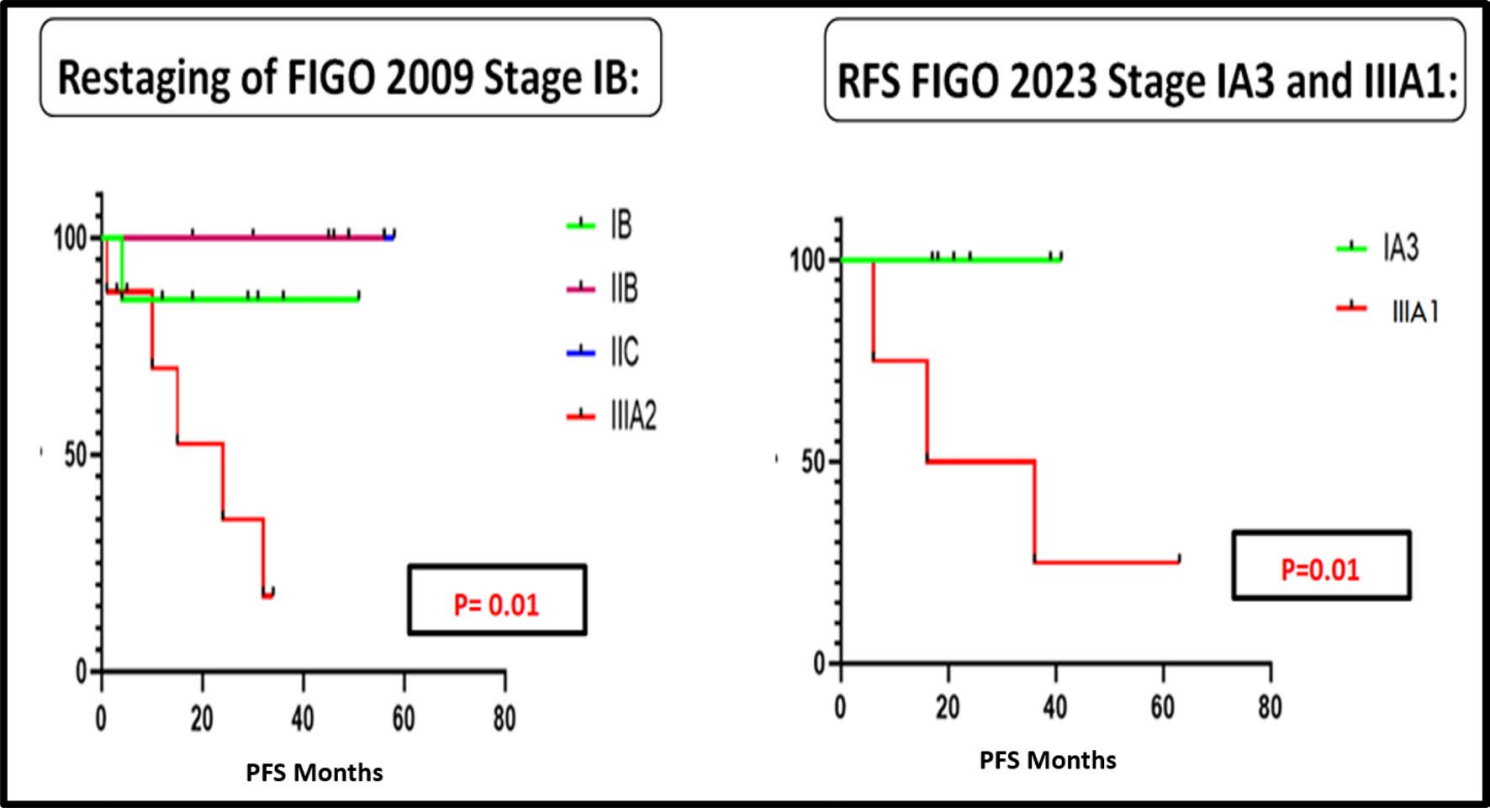
The PFS curves for the FIGO 2009 endometrial staging system when reclassified as per the FIGO 2023 staging system for Stage IB and IIIA

When patients were followed up for a median duration of 45 months, restaging of FIGO 2009 Stage IB and Stage IIIA as per the new FIGO 2023 staging system using the Kaplan–Meier analysis was found to be statistically significant for PFS (p 0.01) as shown in Fig. 5. When we compared the other groups the values were not statistically significant.

Figure 5 depicts the PFS curves for the FIGO 2009 endometrial staging system when reclassified as per the FIGO 2023 staging system for Stages IB and IIIA.

## Discussion

FIGO presented their revised staging of endometrial cancer nearly 14 years after their last update in 2009. This staging was different from its predecessor in that it moved from an anatomy-based staging into a prognosis-based staging system and was based on new molecular stratification. This created quite a furore among the medical fraternity concerning several factors (McCluggage 2023). In the subsequent sections, we will analyze the results of our study with respect to the above factors.

In a study done by Kobayashi et al. they observed a stage migration in 23.4% of the patients which included a larger shift in Stage I to Stage II as per the FIGO 2023 system (Kobayashi-Kato et al. 2023). Schwameis et al. showed 27.6% of patients had a stage shift of which 23.6% were upstaged (Schwameis et al. 2023). We observed that 23% of the patients had a stage shift in our study with the majority of the shifts occurring to Stage II as per FIGO 2023 endometrial system.

Molecular classification is not widely available in many parts of the world, especially in Low Middle-Income Countries (LMICs) and POLEmut testing has no defined surrogate IHC marker like MMRd or p53 groups (McCluggage 2023). This adds to the extra pinch of the patient’s wallet in an already economically backward community. This was evident in our study where only two patients could afford the POLE testing out of 32 patients. This also created an issue while analyzing the other patients as we could not rule out the multiple classifiers which account for 10–15% of the population. It is preferred to keep the molecular classification out of the staging system until a day comes when medical resources are equally available all around the globe. This is not to say that molecular classification is not prognostic (Gilks 2013) but it would be prudent to avoid it in staging and reserve it for the prognostic scoring systems. It would be helpful if a collaborative laboratory system is developed in LMIC countries which can reduce the costs involved as the tests would be done in larger numbers making it cheaper for the general population.

The FIGO 2023 staging system being a “heavy-weight” pathology-based system leads to a phenomenon called “stage migration” where the patient with the same disease has a high probability of being accorded a different stage when reviewed at an advanced diagnostic center due to the availability of molecular testing, determination of depth of sub-serosa involvement (Gilks 2013) and use of certain confusing histological parameters like absence of myometrium invasion and ≤ 50% myometrium invasion. It is well known that the depth of myometrium invasion has poor interobserver variation due to irregular endometrial/myometrium interface (Singh 2019; Gilks 2013). These factors lead to difficulty and confusion while assigning the stage of the patient as it keeps changing during the review of the histopathological report. This was evident in our study as we analyzed all the cases of endometrial cancer and whenever in doubt especially concerning subserosal involvement the opinion of two senior oncopathologists was taken. Not all centres can afford to have this luxury. Moreover, there is no definite consensus in terms of absolute distance from serosa to what constitutes as subserosal involvement. This could be a point that would need further clarification in the subsequent updates of the FIGO staging system.

As per the FIGO 2023 staging system the 5-year mortality in the IA3 group was lower when compared to the IIIA1 group (Berek 2023; McCluggage 2023). This was also shown in a study done by Matsuo et al. (Matsuo 2017). We were not able to study the overall survival due to the shorter follow-up duration in our study. However, we observed the PFS was significantly different for the IA3 Stage compared to IIIA1 Stage. We observed a significant difference in PFS when restaging of FIGO 2009 Stage IB was done. Matsuo et al. demonstrated the prognostic significance of the size of lymph nodal metastatic focus and the nature of peritoneal metastases (Matsuo 2017). With adoption of newer technologies such as sentinel node mapping and ultrastaging, micro metastasis are detected which are missed on routine histopathological analysis (Bogani et al. 2019).

We analyzed the possible impact on adjuvant risk stratification according to the new FIGO 2023 staging system based on the latest ESMO risk stratification system which had not been studied prior. It revealed that seven out of eleven (63%) patients who would have needed adjuvant chemotherapy and radiotherapy but had received lesser form of adjuvant therapy and later developed distant recurrences. We postulate that had this group of patients received more radical treatment we could have prevented these recurrences.

The role of molecular analysis has been exemplified in the latest therapeutic trials of the GARNET study and KEYNOTE -775 study which highlighted the beneficial effect of dostarlimab and a combination of pembrolizumab plus lenvtinib, respectively, in the treatment of endometrial cancer (Oaknin et al. 2022; Makker 2022).

However, keeping the above-discussed limitations of costly molecular profiling in mind it is time we explore an upcoming technology of radiomics (Bogani et al. 2022a). Radiomics is a novel technology that uses a large number of quantitative features from radiological images from ultrasound, contrast-enhanced computed tomography(CECT) and magnetic resonance imaging(MRI) using data characterization algorithms (Rizzo et al. 2018; Jong et al. 2019). Correlation of this information with clinical data and molecular profiling can open up a new frontier in the management of endometrial cancer which will be cost-effective and widely available in the long run if proven to be an effective prognostic indicator (Bogani et al. 2020; Leone Roberti Maggiore et al. 2019). Proper use of radio genomics knowledge can reduce the health expenditure on upcoming molecular testing of new molecular targets such as PI3K/Akt/mTOR pathway which are well beyond the reach of the common man (Bogani et al. 2022b).

The merits of this study include the application of the present ESGO 2022 risk stratification system to analyze the impact of the new staging system on adjuvant treatment. An accurate picture of the fallacies of the staging system performance in LMICs was also discussed.

There are important limitations in this study. The retrospective nature of the study combined with the smaller sample size and limited follow-up meant we could not analyze the impact on overall survival. Our data reflect the availability of resources in this part of the world and need not be applicable in other developed areas.

## Conclusion

There are both advantages and drawbacks of the FIGO 2023 endometrial staging system. While it serves as a radical shift to the prognostic implication of the staging system it still has grey areas which can be resolved through proper appraisal at the global community level to ensure its acceptability to the wider scientific community. Hence we recommend universal adoption of the FIGO 2023 endometrial staging system as it is prognostic in nature and it should be followed by validation studies of the system from various parts of the globe.

## Data Availability

No datasets were generated or analysed during the current study
